# Supplementary material for: Gas-Phase Fluorination of g-C3N4 for Enhanced Photocatalytic Hydrogen Evolution
Source: Nanomaterials (Basel). 2021 Dec 23;12(1):37. doi: 10.3390/nano12010037 (PMC8746965; doi:10.3390/nano12010037)
Supplement: Supplementary file 1 [file nanomaterials-12-00037-s001.zip › nanomaterials-1509788-supplementary.pdf]

Supplementary Material

Gas-Phase Fluorination of g-C_3N_4 for Enhanced Photocatalytic Hydrogen Evolution

Lidong Sun [1], Yu Li [1,*] and Wei Feng [1,2,*]

[1] School of Materials Science and Engineering, Tianjin University, Tianjin 300072, P. R. China
[2] Key Laboratory of Advanced Ceramics and Machining Technology Ministry of Education, Tianjin 300072, P. R. China
* Correspondence: liyuajde@tju.edu.cn; weifeng@tju.edu.cn

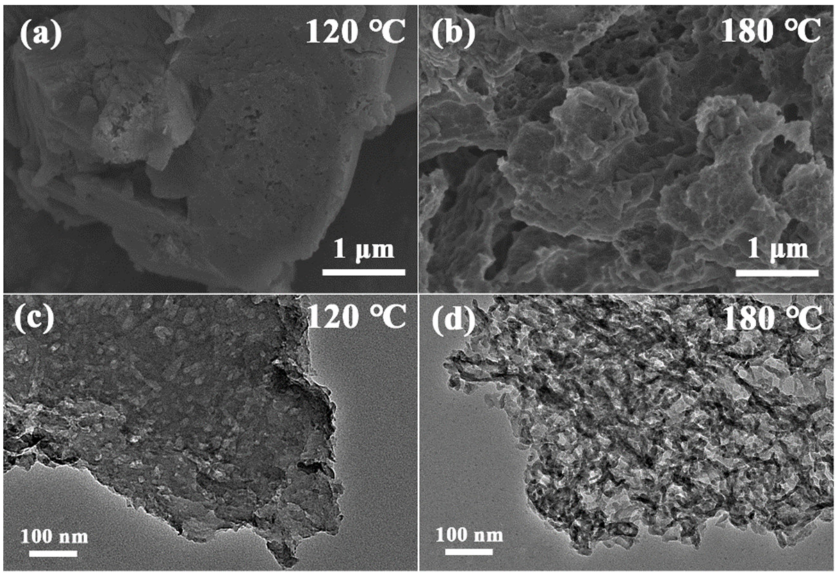

Figure. S1. SEM and TEM images of F-g-CN obtained by treating g-C_3N_4 with F_2 gas at 120 ˚C (a, c) and 180 ˚C (b, d).

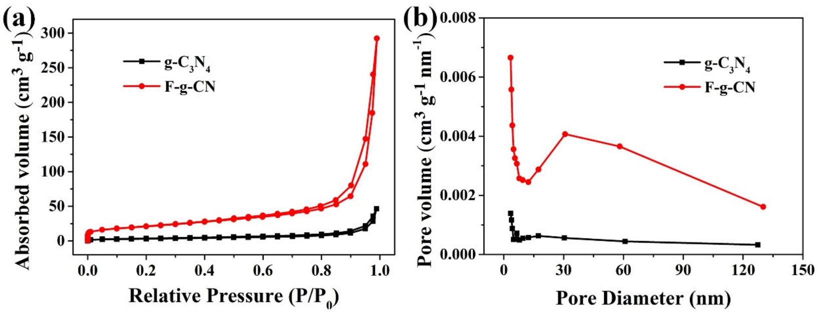

Figure S2. (a) N_2 adsorption/desorption isotherms and (b) the corresponding pore size distribution curves of g-C_3N_4 and F-g-CN.

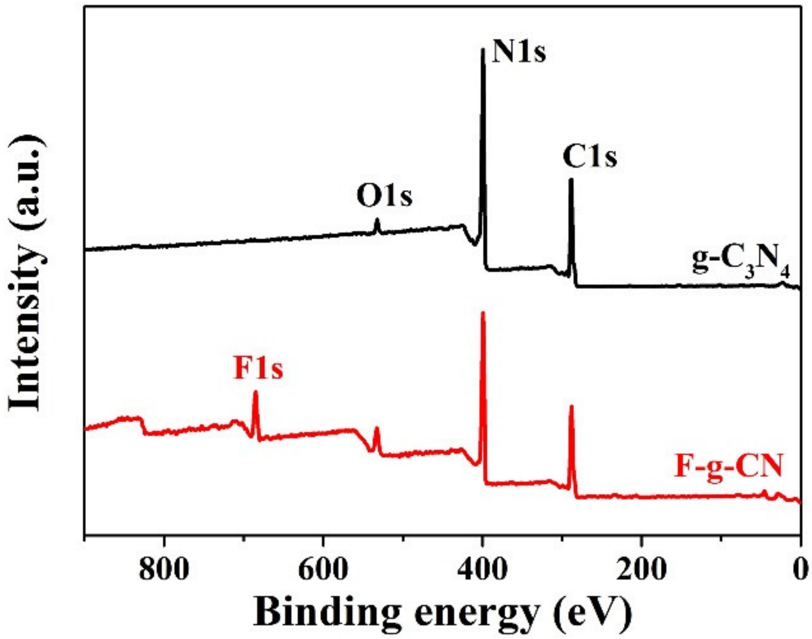

Figure S3. The XPS survey spectra of g-C_3N_4 and F-g-CN.

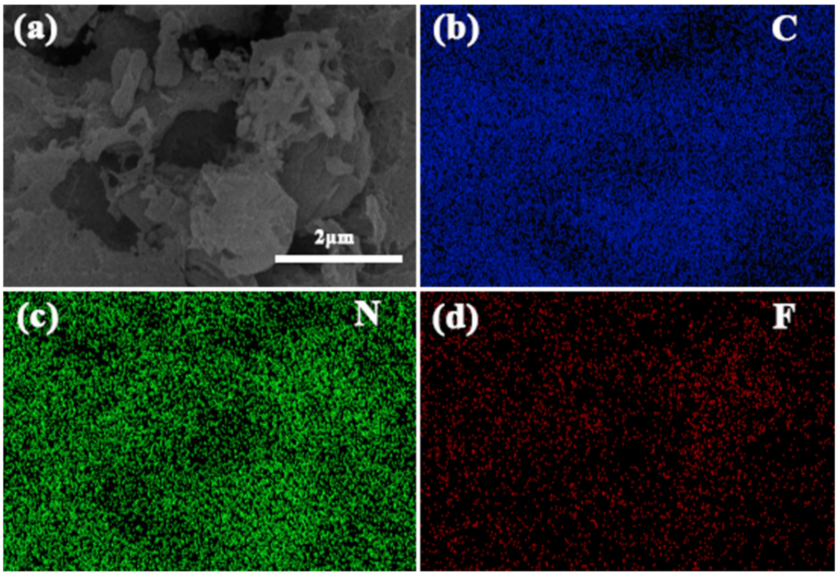

Figure S4. (a) SEM imagine of F-g-CN and (b-d) its corresponding EDS elemental mapping imagines.

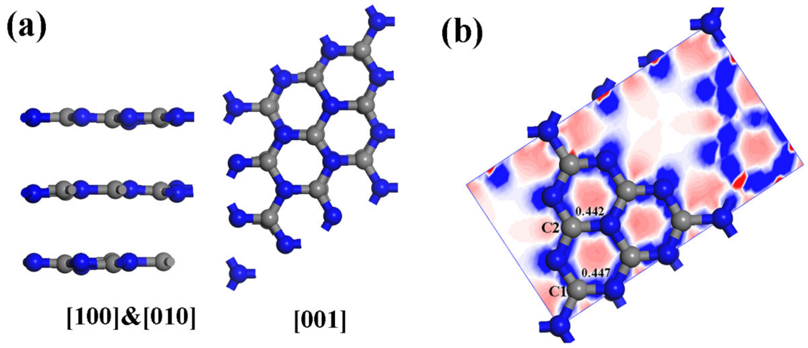

Figure S5. (a) The optimized structure of g-C_3N_4 model viewed from different directions and the blue and grey balls represent nitrogen and carbon atoms, respectively; (b) The corresponding deformation charge density of g-C_3N_4 and the increase/decrease of total electrons density with the density of isolated atoms subtracted is denoted as blue/red, respectively.

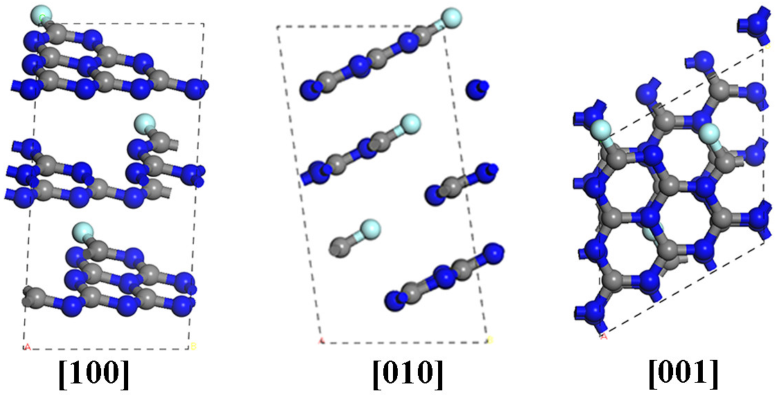

Figure S6. The optimized structure of F-g-CN model viewed from different directions, and the blue, grey and cyan balls represent nitrogen, carbon and fluorine atoms, respectively.

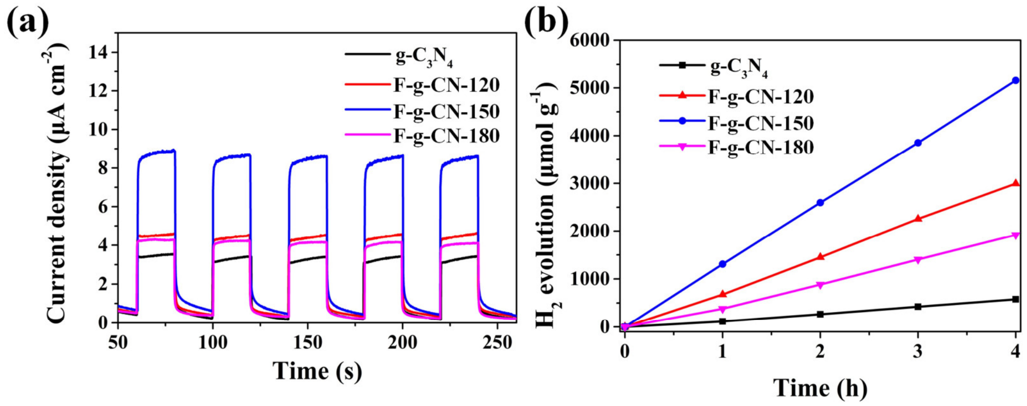

Figure S7. (a) Transient photocurrent responses of g-C_3N_4 and F-g-CN with different fluorinated temperature. (b) Time dependent photocatalytic H_2 evolution over the pristine and the fluorinated g-C_3N_4 samples.

Table S1. The C 1s and N 1s binding energies, the intensity ratio of Nsp^2/Nsp^3 and the atomic ratio of C/N determined from XPS data in the $g\text{-}C_3N_4$ and F-g-CN.

sample	C-F	N=C-N	C-NH	C=C	N-H	N_{3C}	N_{2C}	$N\text{-}sp^2/N\text{-}sp^3$	C/N
$g\text{-}C_3N_4$	N/A	288.4	286.3	284.8	401.2	399.9	398.8	3.34	0.81
F-g-CN	289.5	288.3	286.2	284.7	400.9	400.1	398.8	1.32	0.88

Table S2. The surface atom content of different materials

	C (at. %)	N (at. %)	O (at. %)	F (at. %)
$g\text{-}C_3N_4$	52.10	45.13	2.77	0
F-g-CN-120	46.80	45.24	3.12	3.69
F-g-CN-150	46.31	43.21	3.43	7.05
F-g-CN-180	44.65	39.5	3.93	9.37

Table S3. Different g-C_3N_4 based materials for photocatalytic H_2 evolution.

Materials	Co-catalysts	Light source	Sacrificial agent	H_2 generation [μmol g^{-1} h^{-1}]	AQE [%]	Stability at least [h]	Ref.
2D-C_3N_4	Pd	>400nm	TEOA	1208.6	3.8 (420 nm)	20	[1]
g- C_3N_4	AgPd	>420nm	TEOA	900	-	-	[2]
g- C_3N_4	Au	780 nm >λ>420 nm	TEOA	350.6	1.5 (visible)	13	[3]
Oxygen doped g-C_3N_4	Pt	> 420 nm	TEOA	395.96	1.48 (500 nm)	20	[4]
g-C_3N_4(P)	Pt	≥ 420 nm	TEOA	916.2	6.52 (420 nm)	16	[5]
F-C_3N_4	Pt	>420 nm	TEOA	477.6	2.01 (420 nm)	25	[6]
B doped g-C_3N_4 quantum dots	Pt	>420 nm	TEOA	70.05 μmol h-1	10.0 (420 nm)	20	[7]
CNF	Pt	420-430 nm	-	1167.7	1.7 (420 nm)	25	[8]
g- C_3N_4	NiO	≥ 420 nm	TEOA	68.8	0.01 (500 nm)	30	[9]
g- C_3N_4	Ni_2P	≥ 420 nm	TEOA	474.7	3.2 (435 nm)	20	[10]
F-g-CN	Pt	≥ 420 nm	TEOA	1298	4.53 (420 nm)	20	This work

References

[1] Mo Z.; Xu H.; She X.J.; Song Y.H.; Yan P.C.; Yi J.J.; Zhu X.W.; Lei Y.C.; Yuan S.Q.; Li H. Constructing Pd/2D-C_3N_4 composites for efficient photocatalytic H_2 evolution through nonplasmon-induced bound electrons. *Appl. Surf. Sci.* **2019**, 467, 151-157.

[2] Zou W.X.; Xu L.X.; Pu Y.; Cai H.J.; Wei X.Q.; Luo Y.D; Li L.L.; Gao B.; Wan H.Q.; Dong L. Advantageous interfacial effects of AgPd/g-C_3N_4 for photocatalytic hydrogen evolution: electronic structure and H_2O dissociation. *Chem. Eur. J.* **2019**, 25, 1-8.

[3] Tian H.Y.; Liu X.; Liang Z.Q.; Qiu P.Y.; Qian X.; Cui H.Z.; Tian J. Gold nanorods/g-C_3N_4 heterostructures for plasmon-enhanced photocatalytic H2 evolution in visible and near-infrared light. *J. Colloid Interface Sci.* **2019**, 557, 700-708.

[4] Jiang Y.B.; Sun Z.Z.; Tang C.; Zhou Y.X.; Zeng L.; Huang L.M. Enhancement of photocatalytic hydrogen evolution activity of porous oxygen doped g-C_3N_4 with nitrogen defects induced by changing electron transition. *Appl. Catal. B* **2019**, 240, 30-38.

[5] Wang B.; Cai H.R.; Zhao D.M.; Song, M.; Guo P.H.; Shen S.H.; Li D.S.; Yang S.C. Enhanced photocatalytic hydrogen evolution by partially replaced corner-site C atom with P in g-C3N4. *Appl. Catal. B* **2019**, 244, 486-493.

[6] Ma F.K.; Sun C.L.; Shao Y.L.; Wu Y.Z.; Huang B.B.; Hao X.P. One-step exfoliation and fluorination of g-C_3N_4 nanosheets with enhanced photocatalytic activities. *New J. Chem.* **2017**, 41, 3061.

[7] Wang Y.P.; Li J.L.; Zhao J.L.; Wang J.S.; Li Z.J. g-C_3N_4/B doped g-C_3N_4 quantum dots heterojunction photocatalysts for hydrogen evolution under visible light. *Int. J. Hydrog. Energy* **2019**, 44. 618-628.

[8] Zeng L.; Ding X.; Sun Z.Z; Hua W.M.; Song W.L.; Liu S.Y.; Huang L.M. Enhancement of photocatalytic hydrogen evolution activity of g-C_3N_4 induced by structural distortion via post-fluorination treatment. *Appl. Catal. B-Environ.* **2018**, 227, 276-284.

[9] Liu J.N.; Jia Q.H.; Long J.L.; Wang X.X.; Gao Z.W.; Gu Q. Amorphous NiO as co-catalyst for enhanced visible-light-driven hydrogen generation over g-C_3N_4 photocatalyst. *Appl. Catal. B* **2018**, 222, 35-43.

[10] Zeng D.Q.; Xu W.J.; Ong W. J.; Xu J.; Ren H.; Chen Y.Z.; Zheng H.F.; Peng D.L. To ward noble-metal-free visible-light-driven photocatalytic hydrogen evolution: monodisperse sub–15 nm Ni_2P nanoparticles anchored on porous g-C_3N_4 nanosheets to engineer 0D-2D heterojunction interfaces. *Appl. Catal. B* **2018**, 221, 47-55.
